# Multisensory training improves the development of spatial cognition after sight restoration from congenital cataracts

**DOI:** 10.1016/j.isci.2024.109167

**Published:** 2024-02-09

**Authors:** Irene Senna, Sophia Piller, Chiara Martolini, Elena Cocchi, Monica Gori, Marc O. Ernst

**Affiliations:** 1 Applied Cognitive Psychology, Faculty for Computer Science, Engineering, and Psychology, Ulm University, 89069 Ulm, Germany; 2 Department of Psychology, Liverpool Hope University, Liverpool L16 9JD, UK; 3 Unit for Visually Impaired People (U-VIP), Center for Human Technologies, Fondazione Istituto Italiano di Tecnologia, 16152 Genova, Italy; 4 Istituto David Chiossone per Ciechi ed Ipovedenti ONLUS, 16145 Genova, Italy

**Keywords:** Disability, Neuroscience, Cognitive neuroscience

## Abstract

Spatial cognition and mobility are typically impaired in congenitally blind individuals, as vision usually calibrates space perception by providing the most accurate distal spatial cues. We have previously shown that sight restoration from congenital bilateral cataracts guides the development of more accurate space perception, even when cataract removal occurs years after birth. However, late cataract-treated individuals do not usually reach the performance levels of the typically sighted population. Here, we developed a brief multisensory training that associated audiovisual feedback with body movements. Late cataract-treated participants quickly improved their space representation and mobility, performing as well as typically sighted controls in most tasks. Their improvement was comparable with that of a group of blind participants, who underwent training coupling their movements with auditory feedback alone. These findings suggest that spatial cognition can be enhanced by a training program that strengthens the association between bodily movements and their sensory feedback (either auditory or audiovisual).

## Highlights

• Congenital bilateral cataract children show impaired spatial cognition and mobility
• Spatial cognition improves after sight restoration surgery
• This improvement can be boosted with a 5-day multisensory training
• The training provides augmented audiovisual feedback to participants’ body movements

## Graphical abstract

**Figure:**
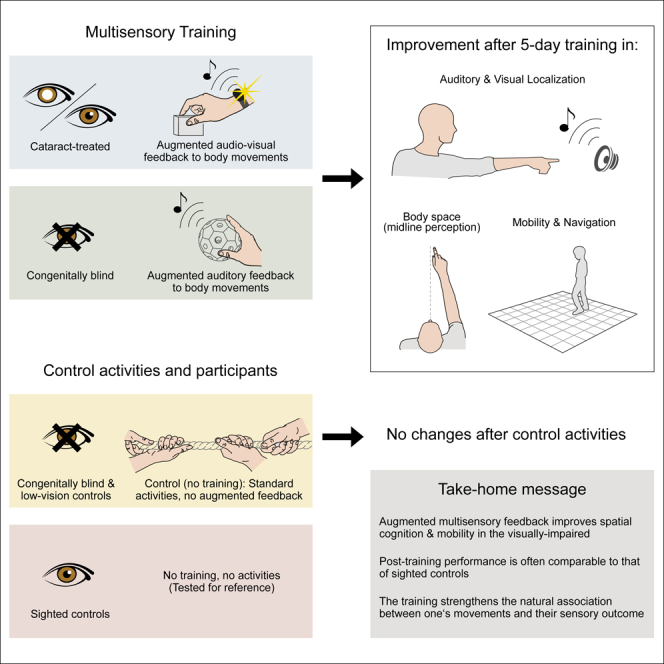

## Introduction

Visual experience provides a crucial contribution to the development of spatial perception and cognition. Evidence of this contribution comes from the case of blindness: visual deprivation early in life usually leads to deficits and delays in the development of spatial and motor abilities. For instance, early blind individuals show impairments in auditory, tactile, and proprioceptive spatial localization in the peripersonal and extrapersonal space.^1^^,^^2^^,^^3^^,^^4^^,^^5^^,^^6^^,^^7^^,^^8^ Tasks requiring the ability to build and update complex auditory or haptic representations of space or use spatial maps for navigation seem particularly challenging.^9^^,^^10^^,^^11^^,^^12^^,^^13^^,^^14^^,^^15^^,^^16^^,^^17^ For instance, in the auditory modality, early blind children and adults fail to understand spatial relationships among different sounds in the environment: this deficit is evident in the auditory spatial bisection task, which requires judging the relative position of a sound within a sequence of consecutive and spatially separated sound sources.^18^^,^^19^^,^^20^^,^^21^ Visually impaired individuals also show poor performance when they are asked to localize single sound sources along the vertical mid-sagittal plane, especially for lower elevations,^4^^,^^7^^,^^8^^,^^22^ or to judge their distance in the far space.^23^^,^^24^ Furthermore, they present delays in the development of motor skills, such as crawling, standing, balancing, reaching and grasping objects, and navigating,^25^^,^^26^^,^^27^ and present balance issues and slower walking speed as compared with sighted controls.^28^

It has been hypothesized that the critical role of vision in the development of spatial abilities is related to cross-sensory calibration processes: as vision typically conveys the most accurate spatial information about the distal environment,^29^ it is a main source used for calibrating the other sensory modalities in processing spatial features.^11^^,^^18^^,^^30^^,^^31^^,^^32^ Consequently, in the case of early-onset visual deprivation, visual calibration of space cannot take place, contributing to deficits in spatial perception and cognition in all sensory modalities. Moreover, as vision often guides target-directed actions and locomotion, the development of motor abilities is also impaired. Support for this cross-calibration hypothesis also comes from animal studies, which report altered auditory spatial maps following absent or distorted visual input.^33^^,^^34^

We have recently provided further support to the cross-calibration hypothesis, by showing that the visual calibration of the auditory space can occur in participants suffering from congenital bilateral cataracts once vision is surgically restored.^22^ The calibration led by the acquired patterned vision occurs even when participants are treated several years after birth, showing that a considerable level of plasticity is maintained even after prolonged early-onset visual impairment.^22^ In particular, the performance of cataract-treated participants in localization and bisection tasks is more accurate and precise than the performance of untreated cataract participants.^22^ This improvement is related to the quality of post-surgical vision, with participants who gained higher visual acuity performing better in the auditory spatial tasks. However, despite such an improvement in auditory space perception, cataract-treated individuals usually do not reach the performance level of the typically sighted controls in the investigated time frame (on average, around 1 year after surgery).^22^ Thus, although some calibration of the auditory space from vision occurs after sight restoration, late cataract-treated participants still present significant impairments in spatial tasks several months or years after cataract removal surgery. We also observed that the mobility of our cataract-treated participants is limited even years after surgery: they navigate in the environment cautiously (cf.^35^), and their actions seem less adept than those of sighted controls. In particular, their grasping and reaching behavior is poor right after surgery: cataract-treated children show more sensorimotor noise than sighted controls, and their ability to recalibrate their sensorimotor system takes years to develop after surgery.^36^ Moreover, cataract-treated participants do not rely on visually predicted object features (such as the estimated weight and size of the to-be-grasped object) for feedforward control of grasping.^37^

In the present study, we investigated whether some brief training can facilitate the development of spatial perceptual and sensorimotor abilities after surgery in late cataract-treated children and adolescents. Previous evidence has reported an advantage of training based on multisensory input over training that relies on one sensory modality alone for perceptual and motor learning^38^^,^^39^^,^^40^^,^^41^^,^^42^^,^^43^^,^^44^ and for recovery following stroke^45^^,^^46^^,^^47^^,^^48^ or sensory impairment and deprivation.^49^^,^^50^^,^^51^^,^^52^^,^^53^ For instance, Gori and colleagues have shown that training procedures relying on the association between auditory, proprioceptive, and motor signals are successful in improving space perception and mobility in both blind and sighted children and adults.^2^^,^^41^^,^^54^^,^^55^^,^^56^^,^^57^^,^^58^ The training procedure proposed by Gori and colleagues relies on a set of motor exercises executed while wearing a device named *Audio Bracelet for Blind Interaction* (*ABBI*), which, once positioned on the participant’s wrist, provides auditory feedback to the participant’s own arm movements.^59^ Although typically sighted individuals mainly acquire spatial competencies via visual-motor associations (i.e., observing the visual consequences of their actions^39^^,^^60^), blind children can use the augmented auditory signal associated with their movements to build a sense of space.^49^ Given that such training strengthens the natural coupling between motor outputs and their sensory feedback, it does not require much attention and cognitive load, resulting in a very intuitive task and thus suitable also for children.^49^^,^^51^

A previous study showed that three months of intensive training with ABBI, reinforcing audio-proprioceptive-motor associations of self-generated movements, induced long-lasting ameliorations of space perception and mobility in a group of blind children.^49^ Given that previous evidence has shown the benefit of including multiple modalities in perceptual and sensorimotor training^45^^,^^49^ and that we found cataract-treated individuals learn to combine vision with other sensory modalities quickly after surgery,^61^ we modified the previous training to also include vision. In other words, instead of using only auditory feedback to participants’ body movements, we also added visual feedback, to provide temporally correlated audiovisual stimulation.

The study aimed to assess whether such training could significantly boost the natural development of spatial, sensory, and sensorimotor abilities that was already taking place—to a certain extent—as a consequence of cataract removal surgery.^22^^,^^36^^,^^61^^,^^62^ To this end, we included in the training a group of Ethiopian children and adolescents who were surgically treated months to years before the beginning of the training. We tested the effect of the audio-visual-motor training on spatial representation and mobility. As in previous studies, we tested participants’ ability to localize sound sources in the peripersonal and extrapersonal space, judge their relative position in space, and reach for them in the environment by walking toward them.^4^^,^^49^ In addition to those tasks, we also explored whether the training could ameliorate the representation of participants’ personal space, given that an appropriate representation of the body’s orientation is essential to navigate the environment and localize external objects in relation to the body.^63^ Finally, as we observed that cataract-treated participants fail to use visual information to scale grip force for grasping,^37^ we investigated whether audio-visual-motor training could support the development of this ability. This was specifically done by including—in the training—activities requiring reaching and manipulating objects.

Importantly, although previous studies in visually impaired individuals have shown that multisensory training can facilitate the development of accurate space perception in non-visual modalities, here we aimed to assess whether such training can lead to improvements also in the visual domain. In a previous study, we showed that visual acuity and the ability to use vision in spatial tasks (i.e., localization of visual stimuli) quickly improve after cataract removal. However, the performance of cataract-treated individuals is still much poorer than that of sighted controls even more than 1 year after surgery.^22^ We here investigated whether the present multisensory training could enhance the ability to localize visual stimuli after surgery. As previous evidence has shown that cataract-treated individuals can use both visual^22^ and auditory information^64^ to recalibrate visual localization, we expect that their visual localization abilities would benefit from training combining audiovisual information.

The training lasted five days for most participants and an additional five days for a subset of participants who had the chance to continue the training for a second session and to be tested in a follow-up 50 days after the end of the training. To assess the effectiveness of the training, we tested the performance of cataract-treated participants before and after the training. Moreover, we compared the performance of cataract-treated participants with that of three other groups in the same age range. First, we included a control group of blind and low-vision participants who did not participate in the training and took part in standard psychomotor activities provided by their school. Second, to evaluate the specific contribution of adding vision to the audio-motor training originally developed by Gori and colleagues,^49^^,^^56^ we compared the effectiveness of the audio-visual-motor training in cataract-treated participants with a purely audio-motor training in a second small group of congenitally blind participants. Third, to assess whether, as a result of the training, participants’ performance could approach or even reach that of the typically sighted population, we compared their post-training outcomes with the performance of a third group of typically sighted participants.

## Results

A total of 18 children and adolescents, surgically treated for congenital bilateral cataracts and tested months or years after surgery (Post-op), and 4 congenitally blind individuals (Blind) took part in a 5-day multisensory training (see STAR Methods, Tables S1 and S2 for details). A subset of participants (six Post-op and all Blind) took part in a further five days of training and were assessed in a follow-up 50 days after the end of the training. A control group for the training included nine blind or low-vision control participants (B&LV) who took part in standard psychomotor activities provided by their school. Finally, we tested around 30 typically developing sighted individuals in each task to assess the performance levels in the typical sighted population, as a reference (Sighted, see STAR Methods for details and Figure S1 for a graphical description of the groups and the training).

The training associated augmented sensory feedback (audiovisual for the Post-op and auditory for the Blind) with participants’ bodily movements. To this end, we used different devices to provide sensory feedback (STAR Methods). For instance, we used a modified version of the ABBI (Audio Bracelet for Blind Interaction), which was originally developed to provide auditory feedback to participants’ arm movements.^49^^,^^59^ By adding a bright red LED to the device, the ABBI provided temporally congruent auditory and visual feedback to participants’ movements. We also relied on other simple devices (e.g., loudspeakers or balls with embedded rattles) that could be used together with visual feedback. All these devices were used with the intent to provide multisensory feedback to participants’ own movements. Participants mainly took part in entertaining group games aimed at improving their spatial representations and their interaction with the environment. The 5-day training involved two sessions per day (one in the morning and one in the afternoon) for each participant, for a total of less than 1.5 h per day (see STAR Methods).

Participants took turns in wearing or holding the device: one participant had a device (e.g., wore the ABBI), whereas the others interacted with the auditory and audiovisual feedback. In this way, the participant with the device could improve their spatial representations through the direct associations between their body movements and the provided feedback. At the same time, the other participants could improve their spatial perception by training their ability to localize and reach auditory or audiovisual targets while interacting with other individuals in the environment.

To assess the effectiveness of the multisensory training, we used a battery of tests, administered twice: right before and right after the training. Most of the tests were previously validated as effective measures of spatial and motor skills in visually impaired individuals and presented a high test-retest reliability.^49^^,^^65^ The tests assess participants’ ability to localize single sound sources (*Auditory localization*), compare the relative positions of a sequence of sounds in space (*Auditory space bisection*), build a spatial representation of the surrounding environment, and navigate in it (*Mobility*). We added new tests to this battery in order to assess participants’ ability to make use of their newly acquired vision to localize visual stimuli (*Visual localization*) and reach and grasp objects (*Grasping*). Finally, we also added a test measuring participants’ personal space perception (*Body midline*).

### Participants taking part in the multisensory training (audio-visual-motor or audio-motor) improved in spatial and mobility tasks

#### Auditory and visual localization

**Figure 1:**
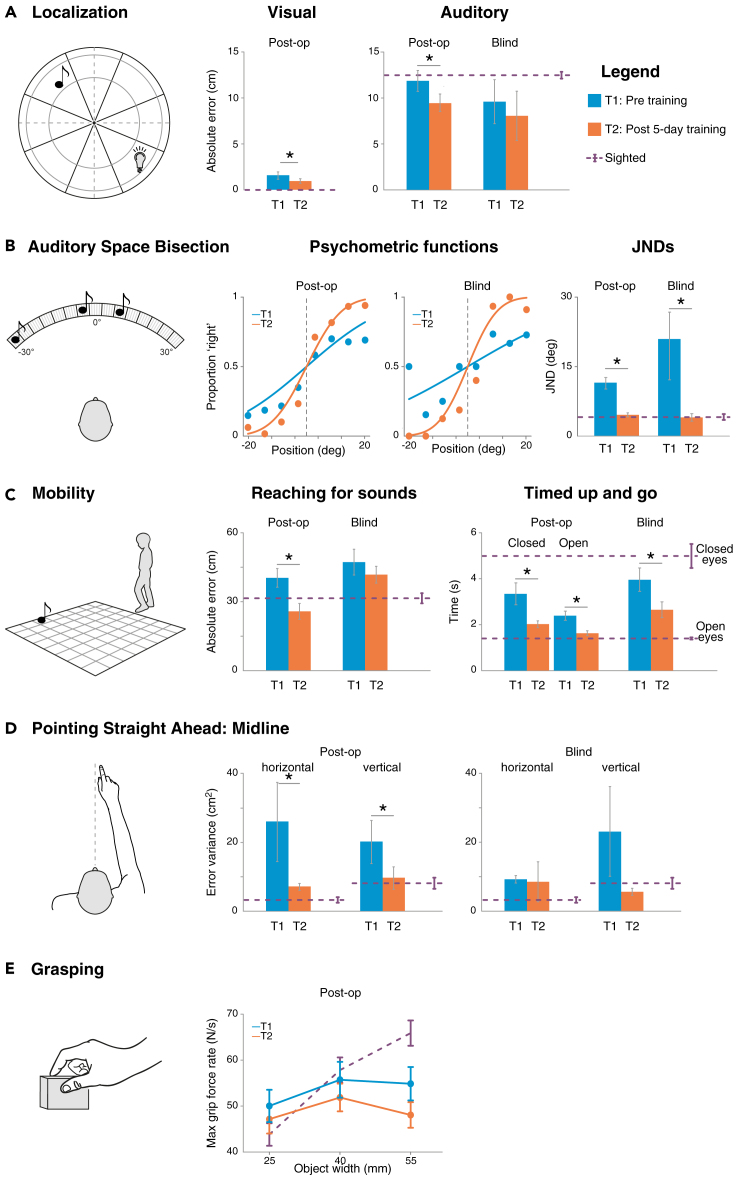
Results of the audiovisual motor training in Post-op and of the audio-motor training in Blind participants (A) Localization of visual and auditory stimuli. Left: setup. All participants localized single sounds, whereas Post-op and Sighted localized also flashes of light in a large setup placed in front of them. Middle and Right: localization error (as the absolute distance between target and participant’s response) in the visual and auditory tasks, averaged across participants in each group and in each session, i.e., before (blue) and after (orange) five days of training (Post-op, N = 18; Blind, N = 4, Sighted, N = 29). (B) Auditory space bisection. Left: setup. Participants were presented with a sequence of three consecutive sounds and reported whether the second sound (i.e., the probe) was spatially closer to the first (left) or third (right) sound. Middle: psychometric functions. Proportions of “closer to the right” responses are plotted as a function of the position of the probe, with negative and positive values indicating probe stimulus closer to the left and the right sound, respectively. Aggregated data, obtained by pulling together the data from all participants, are shown for each group and session. Right: Just Noticeable Differences (JNDs) are shown for each group and session (Post-op, N = 15; Blind, N = 4, Sighted, N = 30). (C) Mobility. Left: setup. In the “Reaching for sounds” task, participants had to localize sounds in the environment and walk toward them. In the “Timed Up and Go task,” they had to perform a brief navigation task as fast as possible. Middle: localization error (as the absolute distance between the position of the target and the location reached by the participant) in the Reaching for sounds task, averaged across participants for each group and session. Right: time needed to perform the Timed Up and Go task, with closed eyes and open eyes, averaged across participants for each group and session (Post-op, closed eyes, N = 18, open eyes, N = 12, Blind = 4, Sighted, N = 34). (D) Body midline representation. Left: procedure. Participants performed a straight-ahead pointing task. Middle and Right: variance of errors (as deviation from the midsagittal plane at the height of the shoulder) along the horizontal and vertical axes, averaged across participants in each group and session (Post-op, N = 18; Blind, N = 4, Sighted, N = 29). (E) Grasping. Left: procedure. Participants grasped equally weighted objects differing in width. Middle: applied forces (measured via a removable six-axis force-torque sensor embedded in the to-be-gasped objects) used to lift each object, averaged across participants in each session and group (Post-op, N = 18, Sighted, N = 22). For each task, bars represent the group average, and error bars represent SEM. The purple dashed lines and bars indicate the mean performance and SEM of the Sighted participants, respectively, shown for reference. Asterisks indicate significant differences (p < 0.05).

This task assessed participants’ ability to localize single sound sources^4^^,^^22^^,^^49^ and light sources^22^ presented on a large circular setup in their frontal plane (Figure 1A, STAR Methods, and cf. ^22^). All participants took part in a block in which they were presented with auditory stimuli. The Post-op and Sighted groups also took part in a block in which they were presented with visual targets. After each stimulus presentation, participants were required to point and reach for the location where they believed the stimulus was displayed on the setup (see STAR Methods). For each trial, we calculated the absolute localization error as the absolute linear distance between the position of the target and that of the participant’s response.^4^^,^^22^

To analyze the effectiveness of the training in the visual block, we fitted the absolute localization error of the Post-op group with a linear mixed effect model (LMM) with the session (pre-training, post-training) as a fixed effect predictor. The model showed that Post-op reduced their visual error between the pre- and posttraining sessions (mean ± standard error, 1.56 ± 0.4 cm vs. 0.96 ± 0.25 cm, respectively, t = 3.69, p = 0.0002, Figure 1A, left). Although Post-op participants improved in localizing visual stimuli, they were still less accurate than Sighted controls, who performed the task perfectly without making any error (0 ± 0, Figure 1A, left).

To analyze participants’ ability to localize sounds before and after the training, for each group (i.e., Post-op, Blind separately), we fitted the absolute localization error in the auditory block with an LMM with the session (pre-training, post-training) as a fixed effect predictor. In the Post-op group, the error was significantly larger in the pre- than in the post-training session (11.86 ± 1.14 cm vs. 9.48 ± 0.96 cm, respectively, t = 3.78, p = 0.0002). In the Blind group, such a reduction of the error between the pre- and the post-training session was only a trend (9.60 ± 2.39 cm vs. 8.06 ± 2.68 cm, t = 1.68, p = 0.09, Figure 1A, middle). However, it is important to consider the low number of participants in the Blind group when interpreting this result, as such a low number affects statistical power (see also the “Limitations of the study” section below). In a second step, we aggregated the data of all participants taking part in the training (i.e., Post-op and Blind together) and compared different LMMs, either including only the session or including also the group (Post-op vs. Blind), alone or in interaction with the session, as fixed effect predictors. The winning model, according to the Akaike information criterion (AIC, STAR Methods), was an LMM including only the session as a fixed effect (i.e., not including the group, alone or in interaction). Although the low number of participants in the Blind group suggests caution when interpreting this result, this finding seems to indicate that both groups participating in the training (Post-op and Blind) reduced their error after the training (t = 4.11, p < 0.0001).

When comparing the performance in the post-training session (Post-op and Blind together) with the performance of the Sighted group via an LMM including group (post-training, Sighted) as a fixed effect, we found that the error shown by participants after the training was significantly lower than that presented by the sighted participants (Sighted: 12.16 ± 0.27 cm, t = 3.21, p = 0.001, Figure 1A, right). Note that, in this and in all other tasks, the analyses yielded consistent results even when comparing the post-training performance of the Post-op group alone (i.e., without including the Blind group) with that of the Sighted group. This result indicated that after the training, participants outperformed the typically sighted participants in sound localization. Before the training, participants’ absolute localization error was comparable with that of the typically sighted, as shown by an LMM on the error with the group (Post-op and Blind pre-training vs. Sighted) as a fixed effect (group: t = 0.91, p = 0.36). In a previous study, we showed that, although after surgery cataract-treated participants reduce their absolute linear error to reach that of the sighted controls, they still present a localization bias along the vertical axis (i.e., a systematic pointing error toward the center of the setup), especially for lower heights.^22^ Here, we found that, after the training, such bias in the auditory modality tended to decrease (see Figure S2, supplemental information). Thus, after the training, cataract-treated participants outperformed sighted controls in localizing sounds in the frontal plane, when considering the absolute error. When looking at their directional error, cataract-treated participants reduced their bias along the vertical axis and no longer significantly differed from sighted controls along the elevation (Figure S2).

#### Auditory space bisection

This task tested participants’ understanding of spatial relationships among spatially separated sound sources.^18^ Participants were presented with three consecutive spatially distributed sounds and had to verbally report whether the second sound was spatially closer to the first or the third one.^18^ The position of the first and third sounds was varied on a trial basis (see STAR Methods), whereas the distance between them was kept constant (covering 40° of visual angle). The second sound (i.e., the probe) was delivered at one of eight possible positions between the other two sounds, varying across trials (see STAR Methods).

For each group participating in the training (i.e., Post-op, Blind, separately), we fitted the probability of responding “closer to the right sound” with a generalized linear mixed model (GLMM) with the position of second sound (i.e., probe’s relative position to the other two sounds), the session (pre-training, post-training), and their interaction as fixed effect predictors. As a measure of the precision in the performance, the GLMM estimated the just noticeable difference (JND) at the 84th percentile (i.e., corresponding to the value of the probe’s location at which the probability of a “closer to the right sound” response is equal to 84%, see^66^ for a description of the approach). The results of the GLMM in the Post-op group showed that participants performed better in the task after the training as compared with before. Indeed, the JND was significantly greater, and thus performance was less precise, in the pre- than in the post-training session (estimated JND ±standard error, 11.41 ± 1.24° vs. 4.59 ± 0.43°, respectively, t = 6.42, p < 0.0001). Similarly, the Blind group reduced their JND after the training (4.04 ± 0.81°), as compared with before (19.45 ± 7.3°, t = 4.1, p < 0.0001, Figure 1B, middle and right panels). Note that, although both groups showed a significant analogous improvement after the training, they tended to differ before the training (t = 1.90, p = 0.058). As reported in our previous study,^22^ cataract-treated participants typically show a better performance in the bisection task than untreated cataract participants and blind individuals. When aggregating the data of all participants taking part in the training (i.e., Post-op and Blind together), the winning model, according to the AIC, was a GLMM including only the position of the second sound, the session, and their interaction as fixed effects (i.e., not also including the group). In other words, both groups significantly performed the task more precisely in the post-training session, as compared with the pre-training session (t = 7.61, p < 0.0001).

When comparing the performance in the post-training session of all participants who took part in the training (Post-op and Blind) with the performance of the sighted controls via a GLMM, including the position of the second sound, the group (post-training, Sighted), and their interaction as fixed effects, we found that the performance of Post-op and Blind participants after the training was comparable with that of the Sighted controls (JND Sighted: 4.19 ± 0.27°, t = 1.32, p = 0.19, Figure 1B, right). This indicates that although before the training participants did not perform as precisely as sighted controls, after the training they improved to reach the levels of their typically sighted peers (Figure 1B).

#### Mobility

In two tasks, we tested participants’ ability to navigate in the environment. In the “Reaching for sounds” task, we tested participants’ ability to localize sounds in the environment and reach for them. In each trial, the experimenter placed a small speaker on the ground, at a desired position, and played a sound (Figure 1C and STAR Methods, cf. ^49^^,^^56^). Once the sound was off, the participants (blindfolded if not blind) had to reach the position from where they believed the sound originated by walking toward it. For each trial, we calculated the error as the absolute linear distance between the position of the target and the location reached by the participant.^49^^,^^56^

We fitted the participants’ absolute errors with an LMM with the session (pre-training, post-training) as a fixed effect predictor. In Post-op, the error was significantly larger in the pre- than in the post-training session (40.37 ± 4.06 cm vs. 25.79 ± 3.46 cm, respectively, t = 5.00, p < 0.0001), indicating that cataract-treated participants improved after the training. Although the Blind group tended, on average, to slightly reduce the error (Figure 1C, middle), such a reduction was far from being significant (47.23 ± 5.62 cm vs. 41.78 ± 3.66 cm, t = 0.79, p = 0.43). However, when aggregating the data of Post-op and Blind, the winning model, according to the AIC, was an LMM including only session as a fixed effect (i.e., not including also group, alone or in interaction; session: t = 4.77, p < 0.0001).

When comparing the performance in the post-training session of all the participants (Post-op and Blind) with the performance of the Sighted group via an LMM including group (post-training, Sighted) as a fixed effect, we found that the error shown by participants after the training was significantly smaller than that shown by the Sighted (33.38 ± 2.97 cm, t = 2.67, p = 0.008).

In the “Timed Up and Go test” task, we assessed participants’ general mobility by testing their ability to orient themselves in the environment and measuring the speed needed to solve a brief navigation task.^49^^,^^56^ At the beginning of each trial, the participants stood blindfolded (if not blind) at a starting position. Upon a go-signal, they started walking until the experimenter touched their shoulder. At that point, they had to walk back to the starting position as fast as possible. All Sighted and a sub-group of Post-op participants (see STAR Methods) performed the test once with closed eyes and once with open eyes. The time needed to perform each trial (from the go-signal to the moment in which the participant reached the starting position) was recorded. We fitted the time (in seconds, log-transformed) needed by participants to conclude the task in each trial with an LMM with the session (pre-training, post-training) as a fixed effect predictor, for each group and task condition (open eyes, closed eyes). In the closed-eyes condition, Post-op became faster in the post- than in the pre-training session (2.02 ± 0.15 s vs. 3.34 ± 0.48 s, respectively, t = 4.95, p < 0.0001). Similarly, the Blind group performed the task faster in the post- than in the pre-training session (2.65 ± 0.35 s vs. 3.95 ± 0.50 s, t = 3.15, p = 0.005, Figure 1C, right). When aggregating the Post-op and Blind data, the winning model was an LMM with only the session as a fixed effect (t = 5.73, p < 0.0001), meaning that both groups showed a similar reduction of the time needed to solve the task. When comparing the post-training performance with that of the Sighted group via an LMM with the group (post-training, Sighted) as a fixed effect, we found that Post-op and Blind after training were faster than Sighted who performed the task with closed eyes (4.99 ± 0.52 s, t = 3.48, p = 0.0006, Figure 1C, right). This outcome results from the fact that sighted participants, unaccustomed to walking without vision, considerably reduced their pace when blindfolded compared with when walking with their eyes open (Figure 1C, right panel). It is worth noting that the performance of the groups in the closed-eyes condition differed already before the training: an LMM on the pretraining performance with the group (Post-op, Blind, Sighted) as a fixed effect predictor showed that Post-op and Blind were both faster than Sighted (t = 5.33, p < 0.0001 and t = 4.57, p < 0.0001, respectively). Moreover, before the training, Post-op were also faster than Blind (t = 2.64, p = 0.009).

In the open-eyes condition, Post-op performed the task faster after the training than before (1.63 ± 0.11 s vs. 2.39 ± 0.20 s, respectively, t = 7.56, p < 0.0001). However, despite significantly reducing the time needed to solve the task, Post-op were still slightly slower than Sighted performing the task with open eyes (1.40 ± 0.05 s, post-training session in Post-op vs. Sighted, t = 2.19, p = 0.03, Figure 1C, right).

#### Body midline

This task evaluated the representation of the perceived egocentric midline, which is essential for body orientation and for localizing external objects relative to the body. We measured the representation of the subjective midline with a straight-ahead pointing task. Blindfolded participants (if not blind) were instructed to perform a series of straight-ahead pointing movements, in front of their perceived body midline at the height of the shoulder (STAR Methods). The experimenter took note of each endpoint pointing position along both the horizontal and vertical axes. In each participant and for each axis (horizontal, vertical), we calculated the variance of the errors, namely the linear distances from the target location (i.e., aligned with the participant’s midsagittal axis and at the height of the shoulder), as a measure of precision. In each group participating in the training, we compared the pre- and post-training variance via Wilcoxon signed-rank tests separately for the horizontal and the vertical axis. After the training, Post-op performed the task significantly more precisely than before. Indeed, they reduced the variance of the error in the post- as compared with the pre-training session along both the horizontal axis (7.03 ± 1.01 cm^2^ vs. 25.88 ± 11.48 cm^2^, respectively, z = 2.11, p = 0.03) and the vertical axis (9.56 ± 3.29 cm^2^ vs. 20.06 ± 6.26 cm^2^, respectively, z = 2.68, p = 0.007, Figure 1D). The Blind group showed an analogous reduction of the variance of the error along the vertical axis in the post- as compared with the pretraining session (5.62 ± 1.01 cm^2^ vs. 23.09 ± 12.97 cm^2^, respectively). However, such a reduction was not significant (p = 0.25), due to the very low statistical power associated with the fact that each subject contributed to the analysis with only one data point, and thus only four values per session entered the analysis for the Blind group. Instead, along the horizontal axis, participants’ performance in the pretraining session was as precise as that in the posttraining session (9.25 ± 1.09 cm^2^ vs. 8.53 ± 5.80 cm^2^, respectively).

We aggregated the data of the post-training session of the two groups (Post-op and Blind), given that their performance did not differ (Wilcoxon rank-sum test, horizontal: z = 0.64, p = 0.52; vertical: z = 0.043, p = 0.96). The posttraining variance of the error of their posttraining session did not differ from that of the Sighted group along the horizontal axis (8.13 ± 1.62 cm^2^, Wilcoxon rank-sum test, z = 0.46, p = 0.66) and the vertical axis (4.06 ± 1.0 cm^2^, z = 1.77, p = 0.08, Figure 1D). This indicates that with the training they improved to reach the levels of the sighted participants.

#### Grasping

This task assessed participants’ feedforward control of grasping, by testing whether they are able to use visual estimations of the to-be-grasped object’s weight in order to scale grip force.^35^^,^^63^^,^^64^ Participants repeatedly grasped and lifted three equally weighted wooden objects differing in size as naturally as possible using a pincer grip. We recorded their applied force to each object via a force-torque sensor inserted at each object’s center (STAR Methods, cf.^37^). Only Post-op and Sighted took part in this task.

We fitted the log-transformed grip force rate in each trial with an LMM with the session (pre-training, post-training), the object width, and their interaction as fixed effect predictors. In Post-op, both the session and the session by width interaction were not significant (t = 0.14, p = 0.89 and t = 0.97, p = 0.33, respectively), meaning that participants did not improve their performance after the training (Figure 1E). The object width was significant (t = 2.57, p = 0.01), which indicated that, at least to some degree, participants were scaling their applied force to the object size. However, they did not do that to the same extent as sighted participants: when comparing the performance of the Post-op group (pre- and post-training aggregated, given that they did not differ) with that of the Sighted group via an LMM with the group (Post-op, Sighted), the object width, and their interaction as fixed effects, the object width by group differed between the two groups (t = 3.40, p = 0.0007). This indicated that Sighted scaled their applied force rate to the object size significantly more than Post-op (Figure 1E), as we have previously shown.^37^

Taken together, the present findings demonstrated that a few days of training providing multisensory experience (either audio-visual-motor or audio-motor) were sufficient to lead to beneficial effects on spatial performance in both the Post-op and Blind groups. The two groups showed similar error reduction in most tasks (Figure 1). In cataract-treated participants, most pre-training abilities were not affected by the amount of visual experience gained in the months to years after surgery (as shown by the lack of significant correlations between pre-training performance in each task and time since surgery in most tasks, Figure S3). In other words, in cataract-treated participants, less than one week of training led to improvements that participants had not shown in the months to years after cataract removal.

### Most improvements were maintained in the follow-up

Details on the second 5-day training session and on the follow-up occurring 50 days after the end of the training are provided in Figure S4 in the supplemental information. Overall, the improvements were maintained for most tasks, with the exception of the *Reaching for sounds* task, where performance in the follow-up went back to pre-training levels in both the Post-op and Blind groups (see supplemental information).

### Blind and low-vision controls who took part in standard psychomotor activities did not show any improvement

**Figure 2:**
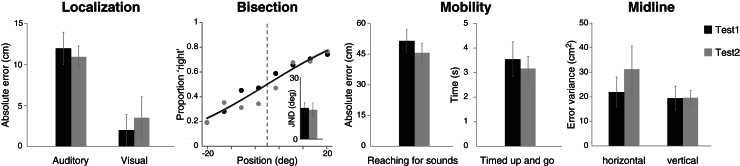
Performance of Blind and low-vision (B&LV) control participants Blind (N = 7) and low-vision (N = 2) control participants took part in each task twice, 5–6 days apart without taking part in the multisensory training (they took part in the standard psychomotor activities provided by their schools). Performance, averaged across participants in each testing session, is shown for each task. All nine control participants took part in each task, except for the visual localization task that was feasible only for the two low-vision individuals. Bars represent group averages for each session (test 1, test 2). Error bars represent SEM.

Blind and low-vision controls (B&LV group) did not show any difference between the performance in the first and second tests (Figure 2). In detail for each task, the test 1 vs. test 2 results are as follows: *Auditory localization*, error: 11.98 ± 1.97 cm vs. 10.89 ± 1.27 cm, t = 1.29, p = 0.20; *Visual localization* (n = 2 low-vision controls), error: 1.95 ± 1.95 cm vs. 3.50 ± 2.56 cm; *Auditory space Bisection*, JND: 15.15 ± 2.82° vs. 14.31 ± 3.59°, t = 0.23 p = 0.81; *Mobility: Reaching for sounds*, error: 51.48 ± 5.47 cm vs. 45.67 ± 4.69 cm, t = 1.12, p = 0.26; *Timed up and go*, time: 3.54 ± 0.71 s vs. 3.17 ± 0.49 s, t = 0.94, p = 0.35; *Body midline*, variance, horizontal axis: 21.91 ± 6.15 cm^2^ vs. 31.12 ± 9.57 cm^2^, Wilcoxon W = 10, p = 0.16, vertical axis: 19.49 ± 4.86 cm^2^ vs. 19.49 ± 3.11 cm^2^, W = 11, p = 0.20 (Figure 2). These findings ruled out the possibility that the improvement observed in the groups undergoing multisensory training is either non-specific to the training type (i.e., linked to the participation in any activities) or merely related to the familiarity with the evaluation tests, which were repeated in close temporal proximity.

## Discussion

In the present study, we investigated whether a brief audio-visual-motor training can enhance spatial abilities and mobility in individuals who were surgically treated for congenital bilateral cataracts several years after birth. We found improvement in most tasks after just five days of training, whereas no changes occurred in the control group. Such improvement was maintained in most tasks in a subset of participants who had the opportunity to participate in a second 5-day training session and to be tested in a follow-up 50 days after the end of the training. These results confirm and extend previous findings that reported enhanced spatial cognition and mobility in visually impaired children following 12 weeks of multisensory training.^49^ Here, we found that even much shorter training can lead to persistent improvements. Importantly, we found that participants’ performance after less than one week of training often approached or even reached the performance of the typically sighted controls.

In a previous study, we demonstrated that the structured vision gained after late cataract removal surgery leads to the development of some recalibration of the perceived space.^22^ In particular, we showed that cataract-treated participants are better than participants with untreated cataracts and blind participants at localizing single stimuli and bisecting a sequence of sounds.^22^ The current study further validates and expands these findings, revealing that before training, cataract-treated individuals outperformed blind participants not only in the bisection task but also in spatially reaching for sound in their environment. This reaffirms the substantial role of vision in calibrating spatial representation, which persists even after prolonged visual impairment. However, despite such an improvement, our earlier study highlighted that the performance of cataract-treated participants in visual and auditory localization tasks, as well as in an auditory spatial bisection task, did not, on average, reach the level demonstrated by the sighted controls even one year after surgery or more.^22^ In particular, the ability of cataract-treated children to localize single flashes of light was rather poor compared with typically sighted controls. This finding aligns with the fact that although visual acuity recovers quickly and remains generally stable after surgery, it is nonetheless inferior to normative levels.^67^^,^^68^^,^^69^ Here, we showed that less than one week of training was sufficient for participants to significantly improve their ability to localize visual stimuli. Impressively, in the auditory localization and bisection tasks, cataract-treated participants reduced their error to reach the performance shown by sighted controls, and this enhancement was maintained over time.

Participants showed better performance also in mobility tasks, becoming more accurate and precise in reaching for auditory sources in the environment and faster in executing a short navigation task. However, only the benefit of the increased speed (which approached the speed shown by sighted controls) was maintained in the subset of participants tested in the follow-up. Beneficial effects of the training were also observed in the representation of the personal space: cataract-treated participants performed a straight-ahead pointing task more precisely after the training, as compared with before the training. The only task in which we did not see any benefit from the training was the grasping task: before the training, participants did not scale their grip force to the visually estimated size and weight of the object to be grasped as much as the controls did. In a previous study,^37^ we showed that cataract-treated participants did not scale grip force and hand aperture to the object’s estimated size and weight even several months after surgery (on average, nine months after surgery). Here, we found that with more time (and thus experience) from surgery, cataract-treated participants showed some very small signs of force scaling to the object’s size and expected weight. However, their performance was still very far from that shown by sighted controls (cf.^37^). Although we included an activity involving reaching and manipulating objects in the training, participants’ poor performance was unchanged after the training. This finding confirms our previous evidence that prolonged early-onset visual impairment harms the development of the ability to use visually predicted properties of objects for feedforward control of grasping. However, it is important to note that, during the training, participants managed to achieve the action goal in each trial (i.e., reaching and manipulating the target), although they did not do it efficiently (i.e., by scaling grip force to object’s size, as sighted do). Future studies should explore whether training strategies providing error feedback whenever the grasp is not performed efficiently could improve these fine aspects of feedforward control of grasping.

We found significant improvement in most tasks also in the group of blind individuals participating in the training. An obvious difference between the cataract-treated group and the blind group is the absence of vision in the multisensory training of the latter. Although the lack of vision results in less information for perceptual learning for the blind participants, the two groups showed analogous benefits from the training in most tasks. This is the case also for the auditory space bisection task: despite the cataract-treated participants showed a trend for some improvement in this task over time after surgery even before the training (cf.^22^; Figure S3), the cataract-treated and blind groups exhibited comparable improvement following the training, reaching the performance levels of typically sighted participants. Although the small sample size of blind participants warrants caution in interpreting the data, this result suggests that the success of the multisensory training is not dependent on prior visual experience: similar benefits can be obtained also in the case of visual deprivation. The only exception to the comparable outcome in the two groups is the reaching for sounds task, where the cataract-treated group showed greater improvement. Although this finding aligns with evidence showing that vision plays a key role in navigating the environment,^70^ the small number of participants in the blind group does not allow us to draw strong conclusions about this possible difference. Thus, although the results in the blind are only preliminary, overall, the fact that the two groups show similar benefits in most tasks suggests that the integration of auditory, proprioceptive, and motor signals may be enough to enhance the representation of space. In other words, coupling self-generated body movements with auditory feedback is sufficient to efficiently guide the development of spatial abilities, even without the additional contribution of visual feedback. This is in line with previous studies demonstrating that brief audio-motor training impacts auditory localization in the blind (e.g.,^57^). This type of multisensory training offers a clear advantage over most training procedures relying on substitution devices, i.e., devices that convert the absent sensory input into signals accessible to another sensory modality.^51^ Indeed, many sensory substitution devices often require the user to participate in several hours of training to learn to interpret the converted signals (e.g.,^71^^,^^72^^,^^73^^,^^74^^,^^75^^,^^76^). Although recent sensory substitution devices and approaches offer rapid benefits,^50^^,^^52^ a few training sessions are still often needed, and such procedures are typically developed for adult participants, rather than children. An advantage of the multisensory training used in the present study is that it strengthens the natural association between own intentional movements and their sensory outcome (in the form of augmented feedback). This makes it an intuitive and user-friendly task, suitable even for children, which does not require extensive familiarization sessions.^49^^,^^56^^,^^57^

It is surprising to see that only five days of training is enough for our participants to show such a remarkable improvement. Although cataract-treated participants are exposed to multisensory events in the environment, in previous studies we found that they need time for developing some ability to use vision in concert with multisensory information and for producing fine motor control. Such development is often slow, with cataract-treated participants still lagging behind their typically sighted peers months to years after surgery.^22^^,^^36^^,^^37^^,^^61^^,^^62^ The fact that their everyday multisensory experience is not sufficient to fully develop multisensory integration, space representation, and mobility is in line with previous studies in dark- or noise-reared cats, showing that simple exposure to a natural environment after months of sensory deprivation is not enough for these cats to develop enhanced responses to multisensory stimuli at the neural level.^77^ Instead, sensory training consisting just of repeated exposure to spatiotemporally congruent audiovisual stimuli leads to the development of multisensory enhanced capabilities in these cats in just a few weeks.^78^ These findings show that the multisensory experience needs to be congruent, consistent, and repeated in order to be effective. In addition to this aspect, in our training, such multisensory information (involving auditory, visual, and proprioceptive inputs) is systematically correlated with the participant’s voluntary bodily movements. We can speculate that the participants can also benefit from the use of the efferent copy generated by their voluntary movements and from the sense of agency derived from this process. The efferent copy provides the input to a forward internal model, used to generate the predicted multisensory feedback, resulting from the motor command.

In summary, we found that less than one week of training was sufficient to develop spatial and motor abilities analogous to those shown by typically sighted controls. Importantly, the children themselves, their caregivers, and the teachers in the schools for the blind reported an improvement in the children’s quality of life, everyday activities, and social interactions. As the training consisted mainly of group activities in the form of games, thus presenting an important social component, it was beneficial also to participants’ social skills. The fact that social interactions benefitted from the training is particularly important, as visually impaired individuals often have limited interactions with others.^51^ Finally, as we did not want this successful training to be just a unique event, we instructed the teachers in the two schools for the blind in Ethiopia where we conducted the training. Although we did not leave the multisensory ABBI there (as it was still a prototype), we donated all the other multisensory devices that we used in the training, so that the teachers could go on with the training also in our absence. In this way, these multisensory exercises became part of the weekly activities systematically provided by their schools to all their visually impaired pupils. This allows us to reach a much larger community than the one originally included in our study. Moreover, as these activities are in the form of entertaining group games, children spontaneously engage in them during their recreational time, hence increasing their learning opportunities.

### Limitations of the study

The present study had a limited number of participants. Participants who got treated for congenital bilateral cataracts several years after birth are extremely rare, thus the sample size in our study was determined by the availability of participants suffering from this rare condition. We included in the training all the available participants we could find over the years of the runtime of the project (N = 18). Ideally, a better control group for the multisensory training should have included a group of cataract-treated participants not taking part in our training (i.e., participating in the control activities). However, we decided to include all the cataract-treated participants in the experimental group (audio-visual-motor training), rather than splitting them into two groups (experimental training vs. control activities) for two reasons. The first one is that the total number of cataract-treated participants was small and that the group was heterogeneous in terms of age at test, age at surgery, visual acuity, and time from surgery. Therefore, we preferred not to split the sample into two rather small groups. The second and more important reason is that we wanted to offer the opportunity to all our cataract-treated participants to participate in the training, rather than exclude some of them from the only occasion they got—so far—to receive a targeted training procedure to improve their condition. For this reason, we included all cataract-treated participants in the training group and a control group of blind and low-vision participants who were already involved in standard psychomotor activities provided by their school. Future studies would benefit from considering a control group involving low-vision participants whose characteristics closely resemble those of our cataract-treated group. For instance, a same-size group of low-vision participants individually matched for age and visual acuity to our cataract-treated cohort could serve as a more suitable control.

To isolate the contribution of vision during the multisensory training, we involved also a small group of blind individuals. It is important to consider the low number of participants in this group when interpreting the results, as such a low number affects statistical power. Future studies involving more participants are needed to be able to draw definitive conclusions about the effectiveness of the training in blind individuals. However, the fact that our blind participants showed the same improvement profile as the cataract-treated participants is promising. In particular, this finding hints at the fact that much shorter multisensory training than that described in^49^ may be already effective, and future studies should be designed to explicitly test this point.

Another limit of the study was that, due to the worsening of the political situation in Ethiopia and the COVID-19 pandemic, we did not have the chance to organize a second trip to Ethiopia in 2020/21 to conduct a second 5-day training session with some of the participants and to retest all of them in the follow-up. For the same reason, we could not extend the audio-motor training to a larger group of blind participants, as initially planned.

## STAR★Methods

### Key resources table

**Table:** 

REAGENT or RESOURCE	SOURCE	IDENTIFIER
**Deposited data**		

Dataset with all experimental results	Mendeley Data	https://doi.org/10.17632/mf48nrdf55.1

**Software and algorithms**		

MATLAB R2021a	MathWorks	https://www.mathworks.com/

### Resource availability

#### Lead contact

Further information and requests for resources should be directed and will be fulfilled by the Lead Contact, Irene Senna (sennai@hope.ac.uk).

#### Materials availability

This study did not generate new unique reagents.

#### Data and code availability

• Full datasets including all the experimental results of each test have been deposited on Mendeley: https://doi.org/10.17632/mf48nrdf55.1 and are publicly available as of the date of publication.
• This study did not generate unique codes.
• Any additional information required to reanalyze the data reported in this paper is available from the lead contact upon request.

### Experimental model and study participant details

#### Participants

Eighteen Ethiopian children and adolescents surgically treated for congenital dense bilateral cataracts (Post-op, sex: 10/8 females/males, 1 left-handed, mean age: 12.21 y, age range: 9-16 y, visual acuity tested right before the training: 4.94 cycles per degree, cpd, range: 0.83-11.56 cpd, time since surgery: 1.48 y, range: 5.16 months–4.13 years) and 4 Ethiopian congenitally blind individuals (Blind, 2/2 f/m, all right-handed, mean age: 12 y, range: 11-13 y) took part in a 5-days multisensory training (see Procedure). Post-op participants presented light perception before surgery (see Tables S1 and S2 for details on the individual participants).

We included a control group of 9 blind and low-vision participants who took part in control activities (see Procedure) and were tested twice, with a 5-6 days interval, without taking part in the multisensory training (B&LV, 6/3 f/m, all right-handed, mean age: 11.02 y, range: 7-16 y). Seven participants in this control group were congenitally blind, 1 had severe low vision, and 1 was partially blind and had spared light perception (Table S1). Five of them were Ethiopian (all blind) and 4 were Italian (2 blind, 2 low-vision). We aggregated the data of the Ethiopian and Italian controls due to overlapping 95% confidence intervals among them in all tested measures.

Finally, we included a group of 89 sighted participants in a similar age range (Sighted). This was done to assess whether, after the training, the participant’s performance reached the performance levels exhibited by the typically developing sighted population. The participants in this group were either German or Ethiopian: since their performance did not differ, the data of the two samples was aggregated. Out of the total number of 89 sighted participants, 29 Sighted (all German, 23/6 f/m, 2 left-handed, mean age: 13.1 y, range: 9-16 y) took part in the *Auditory and visual localization* task (see next paragraph), 30 Sighted (21 German, 9 Ethiopian, 12/18 f/m, 2 left-handed, mean age: 12.38 y, range: 8-17 y) in the *Auditory space bisection* task, 28 Sighted (21 German, 7 Ethiopian, 20/8 f/m, 1 left-handed, mean age: 13.76 y, range: 9-16 y) in the *Body midline* task, 34 Sighted (all German, 30/4 f/m, 4 left-handed, mean age: 14.57 y, range: 13-16 y) in the *Mobility* tasks and 22 Sighted (all German, 9/13 f/m, all right-handed, mean age: 12.78 y, range: 7-21 y) in the *Grasping* task. Around half of the Sighted participants participated in at least two of the abovementioned tasks (see Figure S1 for a graphical description of the study and a summary of the composition of the groups). Participants with cataracts received an ophthalmological examination and underwent cataract removal surgery at the Hawassa Referral Hospital, Ethiopia. Ethiopian participants performed the tasks at the Shashamane Catholic School for the blind or at the Sebeta Blind School. We tested German participants in several schools in the southwest of Germany. We tested blind and low-vision Italian controls at the Istituto David Chiossone for blind and low-vision people, Genova, Italy. The study was approved by the ethics committee of the University of Bielefeld, Germany (EUB 2015-139), Hawassa University, Ethiopia, the Italian Institute of Technology (IIT) and ASL3, Genova, Italy. Participants, or their parents or legal guardians in the case of minors, gave their written consent to participate in the study. A subset of the data relative to the pre-training session of the Post-op and Blind participants and to the performance of the Sighted controls in the *Auditory and visual localization* and *Auditory space bisection* tasks was previously published in.^22^

### Method details

#### Procedure

For the Post-op and Blind participants taking part in the multisensory training, the procedure consisted of three phases: pre-evaluation assessment, multisensory training (audio-visual-motor for the Post-op group and audio-motor for the Blind), and post-evaluation assessment. The training lasted 5 days. To assess the effectiveness of the training, we tested several spatial abilities of the participants right before and right after the training via a battery of tests. The tests were administered on the first day of the training, right before the training started, and then repeated right after the training. Thus, all participants took part in the evaluation tests twice, at 5-6 days distance. Similarly, the B&LV control participants took part in the same assessment twice, 5-6 days apart. The control participants took part in the standard psychomotor lessons provided by the schools for the blind in which they and Post-op participants lived or by the institute caring for them (cf.^49^).

A subset of participants (6 Post-op, and all Blind) took part in a further 5-days of training, and was tested again at the end of this second session and in a follow-up taking place 50 days after the end of the training to check for the stability of any possible improvements. Due to the worsening of the political situation in Ethiopia and the Covid-19 pandemic, we did not have the opportunity to include all participants in the second session of training and in the follow-up.

#### Multisensory training

The training relied on the use of sensory feedback (auditory for the Blind group, and audio-visual for the Post-op group) to provide spatial information about self-motion (cf.^4^^,^^49^^,^^56^). To this end, we used different devices. First, we used a modified version of the ABBI (Audio Bracelet for Blind Interaction), which was originally developed to produce auditory feedback to arm movements.^49^^,^^56^^,^^57^^,^^59^ Being equipped with motion sensors and an audio system, the bracelet starts making sounds as soon as the participant who is wearing the device moves. In the present study, we added a red LED to the bracelet, in addition to a sound (a pure tone of 500 Hz and 180 bpm). The light and the sound were temporally correlated and delivered with the same frequency. We provided this type of stimulation as previous evidence found that concurrent audio-visual feedback (with the same spatiotemporal characteristics) can improve localization, probably via mechanisms of multisensory enhancement involving the superior colliculus (e.g.^45^). In the training, we also used other simple devices providing auditory feedback, such as a loudspeaker and a ball with rattles inside.

The training lasted less than 1.5 hours/day per participant and mainly involved group activities. Each participant took part in two training sessions per day: the first session took place in the morning and lasted 45 minutes, with each group including around 6-7 participants. The second one, in the afternoon, lasted 30 minutes and involved smaller groups of 2-3 participants. In addition to group activities, each person participated in an individual session lasting around 5-10 minutes per day. We chose to form groups with different numbers of participants and to include also individual activities in order to make sure that each participant would actively participate in all the activities. Participants were sorted into groups based on their “level of activity”, with individuals who tended to be more participative and active being included in the same groups, and participants who appeared to be less active in other groups. Creating larger groups allowed to have more entertaining, active, and socially interactive activities, while having smaller groups allowed the opportunity to grant a higher level of participation to each participant. All these strategies were adopted to guarantee that all participants would have around the same amount of experience and exposure to the multisensory stimulation.

In each of the group activities described below, participants took turns wearing or holding the device while interacting with each other. The following activities, mainly in the form of entertaining group games, were either adapted from previous studies^49^ (“*Spatial localization in the environment*”, “*Localization and interactions with trajectories in motion*”) or introduced in the present study (“*Reaching and manipulation of objects*”). To ensure that Post-op participants attended to both the auditory and visual modalities, we sometimes blindfolded them while participating in the activities, to force them to rely on audition. All participants were blindfolded a few times during each session, for a few minutes at a time (i.e., so that, when taking part in one specific activity, among those described below, they would do so with open eyes in some trials, and closed eyes in other trials). The different activities aimed to improve spatial representations and promote locomotion in the environment through interactions with other participants and with objects. The activities described below involved interacting with and reaching for targets in both the peripersonal and the extrapersonal space (i.e., out of reaching), where the localization of sound sources is particularly poor in visually-impaired participants.^23^^,^^24^ Each training session includes all the following activities:

##### Spatial localization in the environment

In different tasks, participants were required to localize a stimulus either in their extra-personal or peri-personal space. For instance, in one task, a group of 2-3 participants sat at the table with one participant wearing the ABBI and sitting on one side of the table and the other two sitting on the opposite side. The participant wearing the ABBI moved the arm freely for a few seconds. As soon as the participant stopped moving (and thus the audio-visual feedback provided by the ABBI stopped), the other participant(s) had to reach for the ABBI (by grabbing it) as soon as possible. This task allowed us to train localization abilities in the peripersonal space, especially in the frontal plane and along elevation. In another task, one participant held the ball with the rattles or the speaker (providing a metronome sound) and walked freely in the environment. As soon as the participants stopped and the auditory stimulus stopped, the other participants had to run and reach for the ball/speaker as soon as possible. The first who arrived took the ball/speaker and it was their turn to move with the device. In other tasks, they had to reach for a static sound source as fast as possible. The experimenter made sure that each participant would wear/hold the device at least once for each activity.

##### Localization and interactions with trajectories in motion

In a series of tasks, participants were required to localize and interact with moving audio-visual stimuli rather than localize static stimuli or estimate the ending point of their trajectory once they stopped, as in the examples above. For instance, in one task one participant held a device and ran around with it. The others had to run after him/her and intercept the device online as soon as possible by grabbing it. Participants took turns carrying the device. Post-op participants carrying the device were allowed to open their eyes, while the others, who had to intercept it, were blindfolded. In another task, participants were asked to stay in a circle and pass the rattling ball to each other either by kicking it with their foot or throwing it with their hands.

##### Reaching and manipulation of objects

This individual activity involved the manipulation and the interaction with objects, rather than with the other participants. This activity was included because in a previous study we found that the ability to program grasping in a feedforward manner based on visual cues (i.e., by scaling grip force and hand aperture to the object’s visual size and visually estimated weight) is impaired after cataract removal surgery.^37^ Given the nature and the aim of this activity, only Post-op participants (i.e., not Blind) took part in it. During this activity, the participant was sitting at the table and was presented with different objects and had to reach for them and move them to new locations. One Post-op participant at the time wore the ABBI and sat in front of a setup consisting of a board (45 by 30 cm) with five embedded circular plates (7.5 cm diameter) that could light up by means of LEDs placed below them. Four objects (differing in shape, size and weight) were placed on top of the circular plates, one on top of each plate, while one plate was left free. At the beginning of each trial, one of the plates below the objects lightened up and the participant had to grasp the object on top of it and move it to the plate that was left free, as fast as possible.

#### Control training

The B&LV control participants took part in the standard psychomotor activities provided by their schools (cf.^49^). These programs aim at the development of fine and gross motor skills, by integrating locomotion activities and motor exercises designed to enhance balance, orientation, coordination, and social interaction, using the unimpaired senses. Furthermore, sports are adapted to accommodate the visually impaired; for instance, interactive games involve children running while holding onto a rope held by others, promoting inclusive participation. Children participate also in musicotherapy and dance sessions.

#### Pre- and post-training evaluation assessment

We used a battery of tests to assess the effectiveness of the multisensory training. Most of the tests were previously validated as effective measures of spatial and motor skills in visually impaired individuals and present a high test-retest reliability.^49^^,^^65^ The tests assess participants’ ability to localize single sounds, compare the relative positions of a sequence of sounds in space, build a spatial representation of the surrounding environment, and navigate in it. We added new tests to this battery to assess participants’ ability to make use of their newly acquired vision to localize or reach and grasp visual stimuli. Finally, we also added a test measuring their personal space perception. The tests were the following:

##### Auditory and visual localization

This task assessed participants’ ability to localize single sound sources^4^^,^^22^^,^^49^ and light sources.^22^ Participants were blindfolded (if not blind) and sat in front of the setup, which consisted of a circular bull’s eye printed on fabric (1 m diameter) hanging from the ceiling, with its center halfway between participants’ eye and ear level (Figure 1A, cf.^22^). All participants took part in a block in which they had to localize auditory stimuli. The Post-op and Sighted groups were presented also with another block in which they were asked to localize visual stimuli. The order of the auditory and visual blocks was counterbalanced across participants. In each trial, the experimenter presented each stimulus by hand directly on the circular setup. In the auditory block, at the beginning of each trial, the experimenter placed a speaker (5 cm per side) on the circular setup at the desired position and played the sound of a metronome (single pulse at 500 Hz, intermittent sound at 180 bpm) for around 3 s (cf.^4^^,^^22^). Then, the speaker was removed. In the visual block, the blindfold was removed, and participants had to keep their eyes closed. At the beginning of each trial, the experimenter positioned a small torch behind the circular setup. When the torch was in the desired position, the experimenter switched it on and asked participants to open their eyes. The light (in the form of a 2 cm diameter disk) was turned off after around 1 s and participants were asked to close their eyes again (cf.^22^). After each stimulus presentation, participants were required to reach for the location where they believed the stimulus was displayed, by directly touching the setup with the index finger of their dominant hand. To make the stimuli easily reachable by each participant, the diameter of the circle in which the stimuli could be presented was 60 cm for the shorter (typically younger) participants, and 90 cm for the taller (typically older) participants, leading to an average diameter of 75 cm (see^22^ for more details).

To help the experimenter cover the whole task space, eight different lines were marked inside the circle, splitting the circle into 8 equally sized sectors (see Figure 1 A, cf.^4^^,^^22^). In each block (auditory and visual condition), the stimuli were delivered in two random positions for each of the 8 sectors, resulting in a total of 16 trials for each block. A numbered grid was printed on the setup, so that the experimenter could note down the number (or combination of numbers) corresponding to the location of the target stimulus and of the participant’s response (endpoint pointing location) for each trial. Such numbers were then converted offline via a table of conversion. For each trial, we calculated the absolute localization error as the absolute linear distance between the position of the target and that of the participant’s response, cf.^4^^,^^22^).

##### Auditory space bisection

By judging their relative position, this task tested participants’ understanding of complex spatial relationships among spatially separated sound sources.^18^ We used the same procedure and stimuli that we used in a previous study involving sighted, cataract-treated, and blind participants.^22^ Participants were blindfolded (if not blind) and sat at a table with their heads comfortably resting on a chin-rest in front of the setup. The setup consisted of a semicircle printed on fabric, where the experimenter manually placed three speakers (5 cm per side, Figure 1B, left panel, cf.^22^). Participants were presented with three consecutive spatially distributed sounds and had to verbally report whether the second sound was spatially closer to the first or the third sound.^18^ The first sound was presented to the participant’s left, the third sound to the participant’s right, while the second sound (i.e., the probe) was delivered at one of 8 possible positions between the two, varying across trials. In each trial, the first and third speakers could be presented in one of four possible combinations, while the distance between them was kept constant (covering 40° of visual angle): -30° and +10°, -10° and +30°, -22° and +18°, -18° and +22°, where negative and positive values indicate locations to the left or right of the participant, respectively (cf.^22^). We varied the position of the first and third sounds to prevent participants from simply reporting whether the probe was either on the left or right side to their midline (i.e., solving the task by relying on egocentric coordinates), as it would happen if the first and third sounds were kept fixed in the same spatial location across trials, at the far left and right of the participant (cf^20^). The three sounds were three animal sounds, displayed one after the other at 500ms intervals: a chicken, a horse and a dog. Each auditory stimulus lasted 2 seconds, in which each animal sound was repeated 3 times. We chose animal sounds (as in^22^), rather than noise bursts or pure tones (as in^18^^,^^19^^,^^20^^,^^21^), to make the task more engaging and easier to understand, especially given that we depended on interpreters for delivering the instructions to participants. After a few practice trials, to familiarize participants with the instructions and the task, participants took part in 2 blocks of 16 trials each. In each block, the probe sound was always the same animal sound (e.g., chicken). In the following block, the probe sound was changed to another one (e.g., dog). We randomized the animal sounds chosen as probes in each block across participants. In each block, the probe was displayed twice for each of the 8 locations, for a total of 32 trials. We have previously verified that results were not affected by the type of animal used as a probe in each specific block (see^22^ for more details). We did not collect the data of three participants of the Post-op group since they failed to understand the task.

##### Mobility

We used two tasks to assess participants’ navigation and mobility. In the “Reaching for sounds” task, we tested participants’ ability to localize sound sources in the environment and reach for them. At the beginning of each trial, participants stood blindfolded (if not blind) in front of the setup, consisting of a 2 x 2 m grid printed on fabric and fixed to the floor (see Figure 1C, cf.^49^^,^^56^ for details). The experimenter placed a small speaker (5 cm per side) on the ground, onto the setup at a desired position, and played the sound (the same used for the Auditory localization task described above) for 5 s. Once the sound was off, the participants had to walk towards the position from where they believed the sound originated and stop where they believed they had reached it. For each participant, the experimenter randomly selected 3 different positions, and each was presented 3 times, for a total of 9 trials. The grid divided the space into 64 squares (0.25 x 0.25 m each, cf.^56^). Each square was numbered so that the experimenter could note down the number (or combination of numbers) corresponding to the position reached by the participant for each trial. Such numbers were then converted offline via a table of conversion. For each trial, we calculated the error as the absolute linear distance between the position of the target and the location reached by the participant (cf.^49^^,^^56^). In the “Timed up and go test” task, we assessed participants’ general mobility by testing their ability to orient themselves in the environment and measuring the speed needed to solve a brief navigation task (cf.^49^^,^^56^). At the beginning of each trial, the participants stood blindfolded (if not blind) in front of the same setup described above. Upon a go-signal (“ready, 3, 2, 1, go”), they started walking on the grid, until the experimenter touched their shoulder. At that point, they had to walk back to the starting position as fast as possible. For each participant, the test was repeated 3 times. In a sub-group of 12 participants, the test was performed once with closed eyes and once with open eyes. Given that we decided to introduce this further condition (i.e., “open eyes”) once the study was already ongoing, we did not have the chance to test all participants in this condition. The Sighted group performed two blocks: one with open eyes and one with closed eyes. The order of the blocks (open eyes, closed eyes) was counterbalanced across participants. The time needed to perform each trial (from the go-signal to the moment in which the participant reached the starting position) was recorded.

##### Body midline

This test assessed the representation of the perceived egocentric midline with a straight-ahead pointing task. Participants stood blindfolded (if not blind) in front of the graduated setup used for the *Auditory and visual localization* task. The centre of the circular setup was aligned with the participant’s midsagittal plane, at the height of the shoulder and arm distance. To have a more precise measurement, we added a measuring tape along the horizontal axis and fixed it onto the setup, with zero at the centre of the setup. Participants were instructed to keep their dominant hand on their chest, at the level of the sternum (starting position) and to point straight ahead, in front of their perceived body midline at the height of the shoulder. After each pointing movement, they returned to the starting position. They performed 10 straight-ahead pointing trials. The experimenter took note of each endpoint pointing position along both the horizontal and vertical axes.

##### Grasping

This task evaluated participants’ ability to use visual cues to estimate the to-be-grasped object’s weight to scale grip force for feedforward grasping control.^37^^,^^79^^,^^80^ At the beginning of each trial, participants sit at the table in front of the setup, with the thumb and index finger of their dominant hand resting in a closed pinch-grip posture on one small screw (starting position) and with the elbow flexed by about 90° (cf.^37^). They were presented with one object at the time, at a comfortable reaching distance (45% of the distance between the acromion and the metacarpophalangeal joint of the middle finger). They were asked to repeatedly grasp and lift one of three equally-weighted wooden objects differing in size (width: 25, 40, and 55 mm, weight: 112 g) as naturally as possible using a pincer grip. They had to lift each object a few centimeters above the table, and go back to the starting position afterwards. We recorded their applied force to lift each object via a removable six-axis force-torque sensor (ATI Mini40) inserted at each object’s center (cf.^37^). Participants took part in 4 blocks of 3 trials each, yielding a total of 12 trials. In each block, the three objects were presented once, in random order. Only Post-op and Sighted took part in this task.

### Quantification and statistical analysis

#### Audio-visual-motor and audio-motor training

To analyze participants’ performance in each task, we proceeded as follows. In the first step, we assessed the effectiveness of the 5 days of training, by comparing the performance of the participants in each group (i.e., Post-op and Blind separately) before the training to their performance at the end of the 5 days of training in each task (i.e., pre- vs post-training session).

In a second step, we compared the two groups taking part in the training (i.e., Post-op vs Blind), to investigate whether they were similarly affected by the training or whether the visual component added to the training would lead to further benefits for the Post-op group. For most tasks (see Results section), this was done by aggregating the results of both groups and comparing models of increasing complexity, either including only the session or including also the group (Post-op vs Blind), alone or in interaction with the session, as fixed effect predictors. The aim was to check whether the results were better explained by aggregating the data of the two groups or by treating them as separate groups.

In a third step, we assessed whether participants’ performance following the training reached the level of the typical sighed population. This was done by comparing the performance in the post-training session in each task (Post-op and Blind aggregated if allowed by the previous analysis) to that of the Sighted group.

Depending on the dependent variable in each task and its distribution (see Results section for details for each task), we either used linear mixed effect models (LMM), generalized linear mixed models (GLMM), or non-parametric statistics to analyze the data. When linear mixed effect models (LMM) were employed, maximum likelihood estimation was used to estimate the parameters. A Probit link function was applied in the case of generalized linear mixed models (GLMM). To account for the heterogeneity among different participants, we included the random intercept as a random effect predictor in each model. When we compared competing models of different complexity, the selection of the best statistical model was based on the Akaike information criterion (AIC^66^^,^^81^), with the best model being the one with the smallest AIC. The assumptions for the linear models were generally met by our dataset in most cases.

#### Training: Additional 5 days + follow-up

To assess whether a further 5 days of training would result in further improvements and whether the effects of the training were maintained over time, we compared the performance of the subset of participants who were tested over time across the four sessions (pre-training, post-first 5 days session, post-second 5 days session, follow-up) for each task. To this end, for most tasks (see Results section for details), we combined the data from both groups (n= 6 Post-op and 4 Blind) and compared models of increasing complexity. These models either included only the session or included also the group (Post-op vs Blind), either alone or in interaction, as fixed effect predictors. This was done to check whether participants’ performance was better explained either by treating Post-op and Blind as two separate groups or by aggregating their data. Results are reported in Figure S4 in the supplemental information.

#### Blind and low-vision controls

To make sure that any possible improvement seen in the training was specifically driven by the training and not by the mere fact that participants were involved in some psychomotor activities, we compared the performance of the B&LV control group in the first and in the second test, 5-6 days apart (test 1 vs test 2) in each task. To this end, for each task, we used the same analyses used to compare the pre- vs post-training session performance of the Post-op and Blind groups.
