# Supplementary material for: Multisensory training improves the development of spatial cognition after sight restoration from congenital cataracts
Source: iScience. 2024 Feb 9;27(3):109167. doi: 10.1016/j.isci.2024.109167 (PMC10897914; doi:10.1016/j.isci.2024.109167)
Supplement: Document S1. Figures S1–S4 and Tables S1 and S2 [file mmc1.pdf]

iScience, Volume *27*

Supplemental information

Multisensory training improves the development of spatial cognition after sight restoration from congenital cataracts

Irene Senna, Sophia Piller, Chiara Martolini, Elena Cocchi, Monica Gori, and Marc O. Ernst

Post-op participants

Subject	Sex	Age (years)	Pre-op visual assessment up to:	Pre-op CSF cutoff (cpd)	Post-op CSF cutoff (cpd)	Time since surgery (y,m,d)
p01	f	14	HM	0.90	1.57	1,5,24
p02	f	9	LP	Unknown	1.56	4,6,17
p03	f	11	FC 50 cm	0.23	1.45	0,5,4
p04	m	14	FC 3m	0.38	11.56	0,5,4
p05	m	11	FC 5m	4.92	9.06	0,5,4
p06	f	10	HM	0.04	3.76	1,1,1
p07	f	11	HM	0.04	10.61	1,0,29
p08	f	9	LP	0.04	0.87	1,0,29
p09	m	17	LP	0.08	3.13	1,5,9
p10	f	17	FC 2m	2.91	7.56	1,5,10
p11	f	12	FC 3m	2.84	4.83	1,5,10
p12	f	10	FC 1m	1.31	8.78	1,5,10
p13	m	17	HM	1.50	0.83	1,5,10
p14	m	11	FC 20 cm	0.60	1.99	1,5,10
p15	m	10	HM	0.71	6.07	1,5,24
p16	f	11	HM	2.03	6.03	1,5,24
p17	m	12	HM	1.89	1.30	1,5,24
p18	m	15	FC 3m	3.40	7.88	3,4,25

Blind participants

Subject	Sex	Age (years)	Group (T=training C=control)	Pathology description	Visual acuity (cpd)
b1	m	12	T	Ocular malformation	None
b2	m	12	T	Ocular malformation	None
b3	f	13	T	Congenital blindness	None
b4	f	11	T	Anophthalmia	None
bc1	m	12	C	Microphthalmia	None
bc2	m	14	C	Congenital blindness	None
bc3	f	12	C	Congenital blindness	None
bc4	f	11	C	Congenital blindness	None
bc5	f	9	C	Congenital blindness	None
bc6	f	16	C	hypothalamic chiasmatic pilocytic astrocytoma (with nystagmus)	4.34
bc7	f	8	C	Retinopathy of prematurity	None
bc8	f	10	C	Congenital bilateral glaucoma	0.68
bc9	m	7	C	Retinopathy of prematurity	None

Table S1. Clinical characteristics of the cataract-treated, blind, and low-vision participants, related to STAR Methods: participants. For the cataract-treated participants, sex assigned at birth, age at test, pre-surgical visual assessment, visual acuity (in cycles per degree, cpd) before and after surgery, and time since surgery at test (in years (y), months (m), days (d)) are reported. Participants' dense bilateral cataracts were classified as congenital, meaning they were either present at birth or developed within the first weeks or months of life [S1]. The diagnosis was based on the participants' families reporting that their children had bright white eyes since birth, and was supported by the fact that all participants showed optical nystagmus, which is considered a signature of early-onset visual deprivation [S2]. Most cataract-treated participants had strabismus and almost half of them had a family history of congenital cataracts, suggesting their congenital cataracts were hereditary (autosomal dominant). Participants were included in the study only for isolated congenital bilateral cataracts (i.e., without further ocular or systemic comorbidity). They underwent a complete ophthalmological evaluation, which included a B-scan ultra-sound ensuring the retina was intact. Prior to surgery, all participants had light perception (*LP*), some perceived hand motion (*HM*), and some could even count fingers (*FC*) up to the specified distance: we report the highest measure participants were able to perform. We tested participants' spatial visual acuity before (when possible) and after surgery by measuring their contrast sensitivity function (CSF) cut-off frequency with the adaptive procedure described in [S3-S5]. According to the pre-surgical test, most participants were classified as suffering from legal blindness or severe low vision. Legal blindness is defined as a visual acuity below 20/400, corresponding to a 1.5 cpd cutoff frequency, according to the taxonomy of the World Health Organization (WHO, ICD, 10th revision), or below 20/200, corresponding to 3 cpd cutoff frequency, according to the guidelines of the National Institute of Health of the United States (NIH). After surgery, participants' visual acuity significantly improved, and many participants transitioned out from the category of legal blindness (pre- vs post-surgery visual acuity, mean ± standard deviation, 1.42 ± 1.42 cpd vs 4.94 ± 3.60 cpd, Wilcoxon signed-rank test, $z = 3.43$, $p = 0.0006$). The post-surgical visual acuity was evaluated the same day on which the pre-training battery of tests was administered. For the congenitally blind and low-vision participants, either involved in the training or in the control group, sex, age at test, description of the pathology, and visual acuity at test (the CSF cutoff frequency in cpd is reported for low-vision participants) are indicated. While some Ethiopian participants were blind due to congenital peripheral damages, we have no medical records regarding the cause of blindness of the remaining participants (for which only 'congenital blindness' is reported). However, their parents or legal guardians reported they had been completely blind since birth. The last 4 blind participants were Italian, all the others Ethiopian.

Task		Post-op		Blind		B&LV controls		Sighted	
		N	Age	N	Age	N	Age	N	Age
**Auditory and visual localization**	5 days training	18	12.2 (2.7)	4	12 (0.7)	9 (2 visuo, 9 audio)	11 (0.7)	29	13.1 (2.3)
	5+5 days&follow-up	6	12.2 (2.4)	4	12 (0.7)				
**Bisection**	5 days training	15	12.3 (2.8)	4	12 (0.7)	9	11 (0.7)	30	12.4 (2.4)
	5+5 days&follow-up	4	12.4 (2.2)	4	12 (0.7)	-	-	-	-
**Mobility**	5 days training	18 open eyes<br>12 closed eyes<br>17 time-up	12.2 (2.7)<br>12.2 (2.9)<br>12.4 (2.6)	4	12 (0.7)	9	11 (0.7)	34	14.6 (1)
	5+5 days&follow-up	5	12.8 (2.1)	4	12 (0.7)	-	-	-	-
**Midline**	5 days training	18	12.2 (2.7)	4	12 (0.7)	9	11 (0.7)	28	13.8 (2.2)
	5+5 days&follow-up	6	12.2 (2.4)	4	12 (0.7)	-	-	-	-
**Grasping**	5 days training	18	12.2 (2.7)	-	-	-	-	22	12.8 (3.8)
	5+5 days&follow-up	6	12.2 (2.4)	-	-	-	-	-	-

Table S2. Participants' number and age for each group and task, related to STAR Methods, Participants. The table shows the number and age (group mean in years (standard deviation)) of the participants in each group, task, and session (i.e., 5-day training for all participants in Post-op and Blind groups and 5 days + additional 5 days + follow-up in a sub-group of participants). Age was comparable across groups in all tasks, except for the Mobility task (Kruskal-Wallis test, Chi-squared = 19.9, $p < 0.001$), where the Sighted were on average slightly but significantly older than Post-op (Bonferroni-corrected Wilcoxon rank sum test $p = 0.02$), Blind ($p = 0.008$), and Blind & Low vision controls ($p = 0.006$).

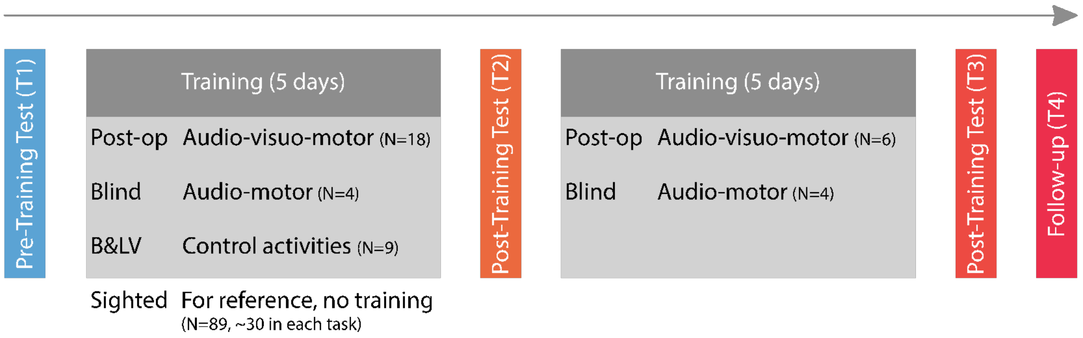

Figure S1. Diagrammatic representation of the timeline of the study and of the compositions of the groups, related to STAR Methods, Participants. Cataract-treated (Post-op group) and blind participants (Blind group) took part in 5 days of multisensory training (audio-visuo-motor and audio-motor, respectively). Blind and low-vision controls (B&LV group) took part in control activities. Participants were tested before and after the training. Typically developing sighted participants (Sighted group) were tested for reference (i.e., for providing information about the performance levels shown by the healthy sighted population). A subset of the participants continued the training for an additional 5-day session and were re-tested after this additional session and in a follow-up taking place 50 days after the end of the training.

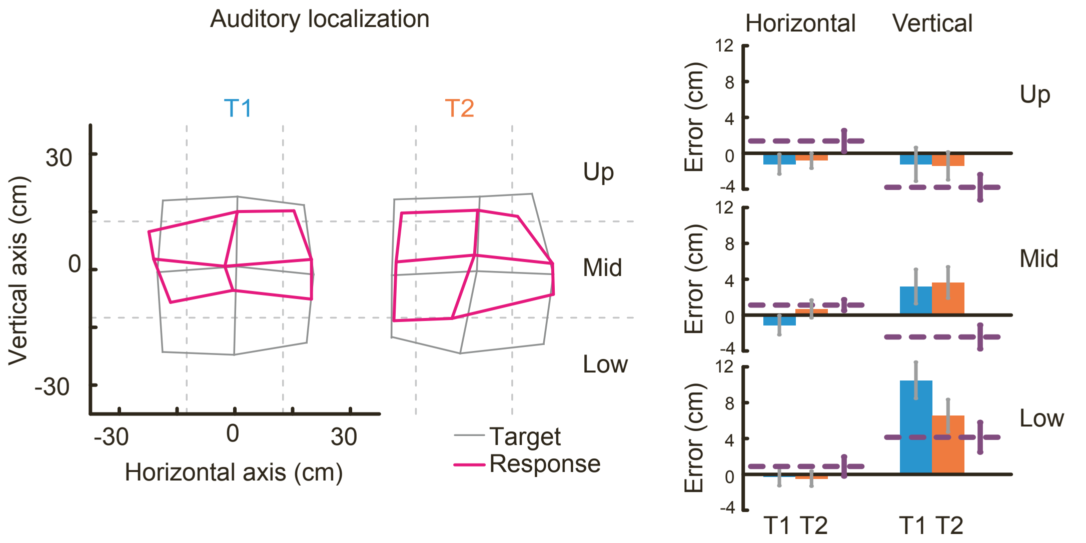

Figure S2. Directional error in the auditory localization task before and after the training in Post-op participants, related to Figure 1 A. Left: pointing performance is summarized in the pre- (T1) and post-training (T2) sessions in the Post-op group by averaging targets' positions and participants' endpoint locations along azimuth (x-axis) and elevation (y-axis) in nine different regions of the set-up (defined by the dashed lines, cf. [S5]). The nine regions result from dividing the setup space into nine equally sized regions: the central region was 25 cm wide and high (i.e., 12.5 cm in each direction from the centre). Given that the target sounds were presented in a 75 cm diameter circle on average across participants, each region was 25 cm wide and high. The grey grid connects the averaged actual targets' locations. The magenta grid connects the averaged position of participants' responses. The horizontal dashed lines indicate the boundaries of the different heights in the analyses: upper (Up), middle (Mid) and lower (Low). Right: directional localization error for the different heights (Up, Mid, Low) along the horizontal and vertical axes in the Post-op group in the pre- (T1) and post-training (T2) sessions. The purple dashed lines and bars indicate the mean performance and SEM of the Sighted control participants. Error bars represent SEM. For each of the two sessions (T1, T2) and for each trial, we calculated the directional error as the difference between the location of the response and that of the target sound along the horizontal axis and along the vertical axis. We divided the analysis into three heights (Up, Mid, Low, cf. [S5]). As the diameter of the target circle in which the stimuli could be presented was 75 cm on average across participants (see STAR Methods), the Mid height was 25 cm (12.5 below/above the centre line), while the Up and Low heights included all target locations above/below that Mid height. For each participant, axis (horizontal, vertical), and height (Up, Mid, Low) we calculated the mean directional error across trials.

Before the training (T1), Post-op participants presented a localization bias along elevation (i.e., a systematic pointing error toward the centre of the set-up), especially for lower heights. This poor localization performance in the lower part of the frontal space, especially along elevation, is in line with previous evidence in blind individuals [S6-S8]. The phenomenon has been attributed to the fact that the visual calibration of the auditory space may be crucial where the contribution of binaural cues is less efficient, as in the case of the vertical mid-sagittal plane [S9]. After the training (T2), Post-op participants reduced such a bias in sound localization. Although such a reduction of the localisation error between the pre-training (mean ± SEM, 10.44 ± 2.08 cm) and the post-training (6.61 ± 1.88 cm) sessions was not statistically significant (Wilcoxon sign rank test, $z = 1.37$, $p = 0.17$), such an error did not differ any longer from that of the Sighted participants (4.14 ± 1.66 cm) after the training (Wilcoxon rank sum test, $z = 0.73$, $p = 0.46$), as it did before the training ($z=2.11$, $p=0.035$). Despite this improvement for the lower heights, Post-op still differed from Sighted in their localisation error in the Mid height in the post-training session (Post-op: 3.64 ± 1.74 cm, Sighted: -2.44 ± 1.33 cm, $z = 2.1$, $p = 0.037$), as they did in the pre-training (3.19 ± 1.91 cm, $z = 2.53$, $p = 0.012$). The error in the Up height was comparable among Post-op in the pre- training (-1.24 ± 1.88 cm) and post-training (-1.41 ± 1.55 cm) sessions and the Sighted participants (-3.79 ± 1.41 cm, all p-values > 0.27). The localization error along the x-axis was comparable among the Post-op participants in the pre- and post-training sessions and the Sighed participants for all heights (p-values > 0.17).

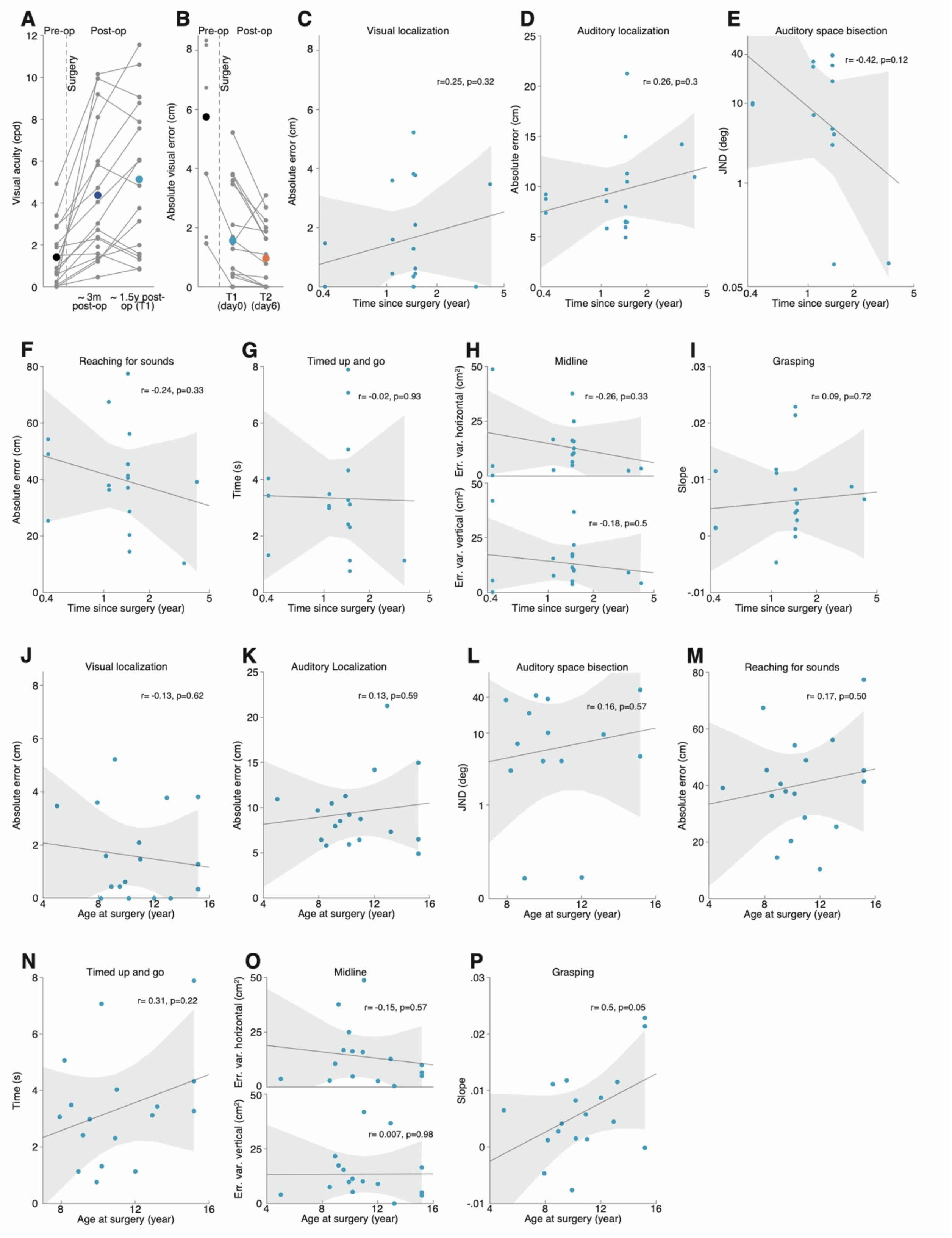

Figure S3. Development of visual acuity and contribution of experience to the development of spatial skills before and after surgery, related to Figure 1. A. Stability of visual acuity following surgery. Participants' visual acuity was tested before surgery and multiple times following surgery. While visual acuity significantly improved after surgery as compared to the pre-surgery evaluation, it was stable after surgery, at the group level:

participants' visual acuity tested right before the training (i.e., on average, 1 year and a half after surgery, range: 5.16 months–4.13 years) did not significantly differ from the visual acuity tested on average 3 months after surgery, meaning around 1 year earlier (range: 4 months- 3 years earlier, Wilcoxon signed-rank test, $z = 1.24$, $p = 0.21$). Individual performance is shown as grey circles, with lines connecting data from the same participant. Coloured larger circles indicate the group's average performance before surgery (black), around 3 months after surgery (dark blue), and at the time of the training, around 1 year and a half after surgery (light blue). B. Performance in the visual localization task (cf. Figure 1 A, left) in a group of participants tested before cataract removal (from [S5], average in black), after surgery before the training (average in light blue), and after the audio-visual-motor training (average in orange). C-I. Correlation between the performance tested in each task before the training and time since surgery (log-transformed). We present these correlations as an indicator of the impact of the amount of post-surgery experience on performance. Typically, performance was not significantly correlated with time since surgery. For instance, the ability to localise visual stimuli improved quickly after surgery (cf. [S5]), but it was stable in the months to year after surgery and before the training. The only task in which performance showed a trend to improve naturally over time after surgery was the auditory spatial bisection (cf. [S5]). However, such improvement happened very slowly, as participants' performance was still far, on average, from the performance level of the sighted controls more than 1 year after surgery (i.e., right before the training). J-P. Correlation between age at surgery and performance in each task. We present these correlations as an indicator of the impact of maturational factors (reflected by age) and of the amount of pre-surgery experience on performance. Age at surgery typically did not correlate with task performance, except for the grasping task, in which older children tended to show greater scaling of the grip force, in accordance with previous studies ([S10]). Correlations considering age at test, rather than age at surgery, lead to analogous results in each task.

In each panel, we report the Pearson correlation coefficient r between time since surgery or age at surgery and performance in that task and the associated p-value. The light-grey shaded area indicates the 95% confidence interval of the regression line.

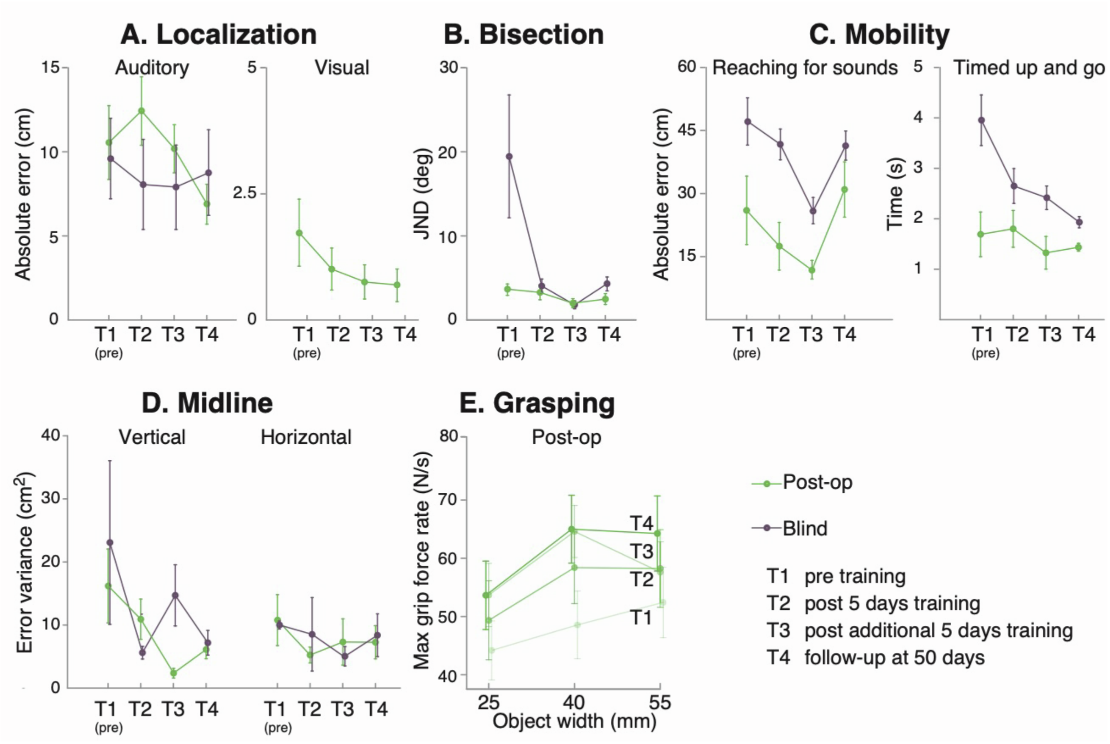

Figure S4. Results of an additional 5-day training session and the follow-up in a subset of Post-op participants and in Blind participants, related to Figure 1. T1: pre-training assessment, T2: tests administered right after the first 5-day training session, T3: evaluation at the end of the second 5-day training session, T4: follow-up 50 days after the end of the second training session. *A. Auditory and visual localization*. We analyzed the error in each trial (linear distance between the target's location and the participant's response) separately for the auditory and visual tasks. In the auditory task, the winning model according to AIC was an LMM including only session (T1 (pre), T2, T3, T4) as a fixed effect predictor: irrespective of the group (Post-op or Blind), participants' error declined in T3, as compared to T2 (9.26 ± 1.29 cm vs 10.68 ± 1.67 cm, respectively, $t = 2.05$, $p = 0.041$, Figure S2A). Note that this differed with what we found in the whole sample (see main text): when considering the whole sample of 18 Post-op participants and not only the 6 retested over time, such an error reduction at the group level occurred already at the end of the first 5 days of training (T2), compared to pre-training (T1). Importantly, participants performed more accurately in the follow-up session (T4, error: 7.63 ± 1.19 cm) than in all the other sessions: T1 (10.17 ± 1.55 cm, $t = 3.44$, $p = 0.0006$), T2 ($t = 4.18$, $p < 0.0001$) and T3 ($t = 2.07$, $p = 0.039$). This finding indicates that the error reduction taking place after the second training session continued with time after the end of the training. In the visual task, a LMM on the error of the Post-op with session as a fixed effect predictor indicated that the error was larger in the pre-training session (T1, 1.73 ± 0.66 cm) as compared to any other session (T2: 1.01 ± 0.42 cm, $t = 2.65$, $p = 0.008$; T3: 0.76 ± 0.34 cm, $t = 3.59$, $p = 0.0004$; T4: 0.69 ± 0.32 cm, $t = 3.84$, $p = 0.0001$). This result indicates that the error in the visual task was reduced in the first 5-day training session and was stable in the following tests.

B. Auditory space bisection. The winning model was a GLMM on the probability of responding "closer to the right sound" with position of second sound, session, and their interaction as fixed effect predictors (i.e., without group). The just noticeable difference (JND), calculated from the GLMM at the 84th percentile, was significantly greater, and thus performance was less precise, in the pre-training (T1: 8.73 ± 1.21 °) than in any other session (T2: 3.79 ± 0.54 °, $t = 4.02$, $p < 0.0001$; T3: 1.99 ± 0.34 °, $t = 4.70$, $p < 0.0001$; T4: 3.50 ± 0.50 °, $t = 4.24$, $p < 0.0001$). After improving already following the first 5 days of training, performance further improved in T3 compared with T2 ($t = 2.72$, $p = 0.007$). In T4, the JND slightly increased compared to T3 ($t = 2.44$, $p = 0.01$), to reach levels comparable to the first post-training session, T2 ($t = 0.43$, $p = 0.67$). Despite the winning model did not include group, from the figure (Figure S2B), it is evident that the reduction of the JND was driven by the Blind group. This happened because, incidentally, the 6 Post-op participants tested over

time were the ones performing better in this task already before the training, within the whole sample of 15 Post-op participants. Indeed, they were already at the performance levels of the Sighted group in the pre-training session.

C. Mobility. In the "Reaching for sounds" task, the winning model was an LMM on the absolute error with both session and group (without their interactions) as fixed effect predictors. Irrespective of the session, the error done by the Post-op was overall smaller than the error in the Blind group (t = 6.51, p < 0.0001, Figure S2C, left). However, both groups showed a similar pattern across the different sessions: they tended to reduce the error in T2 (Post-op: 17.43 ± 5.64 cm, Blind: 41.77 ± 3.66 cm) as compared to the pre-training (T1, Post-op: 26 ± 8.13 cm, Blind: 47.23 ± 5.62 cm, t = 1.89, p = 0.059), and they further reduced the error in T3 (Post op: 11.86 ± 2.19 cm, Blind: 26.01 ± 3.13 cm), which significantly differed from T1 (t = 7.4, p <0.0001) and T2 (t = 2.66, p = 0.008). Unfortunately, this improvement was not maintained in the follow-up, where the error increased to reach pre-training levels in both groups (Post-op: 30.98 ± 6.57 cm, Blind: 41.49 ± 3.46 cm, T4 vs T1, t = 0.06, p = 0.96). The error in T4 showed a trend for being higher than that in T2 (t = 1.95, p = 0.052) and was significantly higher than the error in T3 (t = 4.61, p < 0.0001). These results indicate that participants reduced the error in the first 5 days of training and they further reduced it with an additional 5-days session of training, but they did not maintain such an improvement over time following the end of the training.

In the "Timed up and go test" task (closed eyes condition) the winning model was an LMM on the time needed to conclude the task in each trial with session, group (Post-op, Blind) and their interaction as fixed effect predictors. Overall, Post-op were faster than Blind (Group: t = 5.78, p < 0.0001, Figure S2C, right). This happened because, as it happened for the *auditory space bisection* task, the 6 Post-op tested over time were the fastest ones within the whole sample of 17 Post-op already in the pre-training session. Being already so fast in the pre-training session (1.69 ± 0.44 s), they did not further significantly reduce the time needed to perform the task in the following sessions compared to the pre-training T1 (T2: 1.80 ± 0.37 s, t = 0.25, p = 0.80; T3: 1.33 ± 0.32 s, t = 1.25, p = 0.22, T4: 1.44 ± 0.08 s, t = 0.04, p = 0.97, Figure S2C, right). Instead, the Blind group was significantly slower in the pre-training session (T1, 3.95 ± 0.50 s) than in all other sessions (T2: 2.65 ± 0.35 s, t = 3.28, p = 0.002; T3: 2.42 ± 0.23 s, T4: 1.93 ± 0.11s). Participants became faster already after 5 days of training, and they maintained this speed also the next session of training (T2 vs T3: t = 0.76, p = 0.45). In the follow-up (T4), participants further reduced the time needed to perform the task as compared to T2 (t = 2.34, p = 0.024), while their performance did not significantly differ from that in T3 (t = 1.58, p = 0.12). We did not have data on the open eyes condition, because that condition was introduced in a second step and, unfortunately, we did not manage to test any of the participants included in the follow-up in that condition.

D. Body midline. For each participant, axis (horizontal, vertical), and session, we calculated the variance of the errors, namely the linear distances from the 0 in the 10 trials for each axis. We compared the variance across the different sessions via Friedman tests, separately for the horizontal and the vertical axis. Given that the patterns of results were similar in the two groups (Post-op, Blind), we aggregated their data (Figure S2D). Outliers above or below 2 SD from the group mean for each axis were excluded from the analyses. This led to the exclusion of 2 participants from the analysis on the vertical axis and 1 from that on the horizontal. Results were not significant for both the vertical (Chi-square = 3.15, p = 0.37) and horizontal axis (Chi-square = 2.47, p = 0.48), and the same happens when treating the two groups separately. However, from their average variances in Figure S2D, it is possible to appreciate that, overall, the variance seemed to be reduced in the first two sessions of training. However, while such a reduction appeared to be maintained in the follow-up for the vertical axis, this was not the case for the horizontal, where the variance raised again at pre-training values.

E. Grasping. We fitted the log-transformed grip force rate in each trial with an LMM with the four sessions, object width, and their interactions as fixed effect predictors. Neither session or the session by object width interaction were significant (all p-values > 0.22), indicating that the performance of the Post-op participants was similar in the four sessions (Figure S2E). Moreover, object width was not significant, indicating that participants did not scale their applied force to the object size, as sighted participants typically do. These findings indicate that participant's performance in this task was not affected by the training.

In summary, the subset of the participants that could take part in a second 5-day training session and be tested in a follow-up improved and maintained the improvement in

most tasks, with the exception of the *Reaching for sounds* task, where performance in the follow-up went back to pre-training levels in both the Post-op and Blind groups. Moreover, cataract-treated participants did not show any benefit from the training in the grasping task: they did not show any improvement at the end of the first 5-day training session, and the situation was unchanged after the second 5-day training session and in the follow-up.

Supplemental references

S1. Wu X., Long E., Lin H., Liu Y. (2016). Prevalence and epidemiological characteristics of congenital cataract: a systematic review and meta-analysis. *Sci Rep*, **6**, 1–10. doi: 10.1038/srep28564

S2. Papageorgiou E., McLean R.J., Gottlob I. (2014). Nystagmus in childhood. *Pediatr Neonatol*, **55(5)**, 341–351. doi: 10.1016/j.pedneo.2014.02.007

S3. Senna I., Andres E., McKyton A., Ben-Zion I., Zohary E., Ernst M.O. (2021). Development of multisensory integration following prolonged early-onset visual deprivation. *Curr Biol*, **31(21)**:4879-4885.e6. doi: 10.1016/j.cub.2021.08.060

S4. Senna I., Piller S., Ben-Zion I., Ernst, M.O. (2022). Recalibrating vision-for-action requires years after sight restoration from congenital cataracts, *eLife*, 11:e78734. doi: 10.7554/eLife.78734

S5. Senna I., Piller S., Gori M., Ernst M.O. (2022). The Power of Vision: Calibration of auditory space after sight restoration from congenital cataracts. *Proc R Soc B: Biol Sci*, **289(1984)**:20220768. doi: 10.1098/rspb.2022.0768

S6. Finocchietti, S., Cappagli, G., Gori, M. (2015). Encoding audio motion: spatial impairment in early blind individuals. *Front Psychol*, **6**:1357. doi: 10.3389/fpsyg.2015.01357

S7. Lewald J. (2002). Vertical sound localization in blind humans. *Neuropsychologia*, **40**, 1868–1872. doi: 10.1016/s0028-3932(02)00071-4

S8. Zwiers M.P., Van Opstal A.J., Cruysberg J.R. (2001). A spatial hearing deficit in early-blind humans. *J Neurosci*, **21**: RC142: 1–5. doi: 10.1523/JNEUROSCI.21-09-j0002.2001.

S9. Voss P. (2016). Auditory spatial perception without vision. *Front Psychol*, **7**:1960. doi: 10.3389/fpsyg.2016.01960

S10. Gordon A.M., Forssberg H., Johansson R.S., Eliasson A.C., Westling G. (1992). Development of human precision grip - III. Integration of visual size cues during the programming of isometric forces. *Exp Brain Res*, **90 (2)**, 399–403. doi: 10.1007/BF00227254.
